# Implications of Surface and Bulk Properties of Abutment Implants and Their Degradation in the Health of Periodontal Tissue

**DOI:** 10.3390/ma6125951

**Published:** 2013-12-18

**Authors:** Erica Dorigatti de Avila, Rafael Scaf de Molon, Denise Madalena Palomari Spolidorio, Francisco de Assis Mollo

**Affiliations:** 1 Department of Dental Materials and Prosthodontics, School of Dentistry at Araraquara, Universidade Estadual Paulista—UNESP, Araraquara, São Paulo 14801-903, Brazil; E-Mail: mollo@foar.unesp.br; 2 Department of Diagnostic and Surgery, School of Dentistry at Araraquara, Universidade Estadual Paulista—UNESP, Araraquara, São Paulo 14801-903, Brazil; E-Mail: molon.foar@yahoo.com.br; 3 Department of Physiology and Pathology, School of Dentistry at Araraquara, Universidade Estadual Paulista—UNESP, Araraquara, São Paulo 14801-903, Brazil; E-Mail: dmps@foar.unesp.br

**Keywords:** abutment implants, bacterial adhesion, cell adhesion, titanium, zirconia

## Abstract

The aim of the current review was to investigate the implications of the surface and bulk properties of abutment implants and their degradation in relation to periodontal health. The success of dental implants is no longer a challenge for dentistry. The scientific literature presents several types of implants that are specific for each case. However, in cases of prosthetics components, such as abutments, further research is needed to improve the materials used to avoid bacterial adhesion and enhance contact with epithelial cells. The implanted surfaces of the abutments are composed of chemical elements that may degrade under different temperatures or be damaged by the forces applied onto them. This study showed that the resulting release of such chemical elements could cause inflammation in the periodontal tissue. At the same time, the surface characteristics can be altered, thus favoring biofilm development and further increasing the inflammation. Finally, if not treated, this inflammation can cause the loss of the implant.

## 1. Introduction

Dental implants have achieved great clinical success in the last 20 years. However, late failure due to a disruption between the implant and the mineralized tissues after osseointegration has been established can still occur due to overloading or microbial infection [1,2,3]. While the role of implant surfaces in achieving and maintaining osseointegration has been researched extensively, the second reason for the failure of implants,* i.e.*, the presence of bacterial biofilms on the implant surfaces, has received less attention. Specifically, the main problem of osseointegration has been solved through the use of high-quality implants with appropriate surface treatments and adequate surgical techniques to avoid peri-implant tissue inflammation. However, the biofilm on these surfaces may cause inflammation of the peri-implant mucosa, leading to subsequent destruction of the alveolar bone that is in contact with the implant threads. In addition to sustained osseointegration, good integrity of the peri-implant mucosa at the transmucosal implant surface is another vital factor in long-term implant success.

Experimental results from* in vitro* and* in vivo* studies strongly suggest that some types of surface modifications promote more rapid bacterial and epithelial cell adhesion than machined surfaces. This difference may depend on an altered surface chemistry and/or increased texture at the micrometer scale [4,5]. Studies have also shown that surface characteristics play a special role in the biological performance of abutment implants. The surface properties of interest for abutment implants can broadly be divided into structural properties and chemical properties. Thus, the aim of the current review was to investigate the implications of the surface and bulk properties of abutment implants and their degradation in relation to periodontal health.

## 2. Attention to Prosthetic Components—Abutments

For dental implants to be successful, direct bone-to-implant contact without interposition of any other tissue is needed [6]. At the same time, to preserve osseointegration around dental implants, biocompatible surfaces that are adherent to epithelial cells but non-adherent to bacteria are likewise needed. Patients who have lost teeth due to periodontal disease have periodontal bacteria in their mouths. These bacteria can adhere to other surfaces present in the oral cavity, including restorations, prosthesis and abutment implants. Biofilms that develop on abutment surfaces may cause peri-implantitis. Peri-implantitis is defined as a bacterial infection characterized by inflamed, swollen, and bleeding soft tissues resulting in suppuration and crater-like destruction of the alveolar bone adjacent to a functional implant [7,8]. Because bacterial adhesion and colonization has been implicated as the main causative factor in the initiation and progression of peri-implant disease, the implant and periodontal structures need to be protected from bacterial invasion and subsequent infection [9]. To this end, surfaces that can inhibit bacterial adhesion but are also nontoxic to the periodontal tissue are needed [10]. The response of cells and tissues to foreign bodies depends on the latter’s properties and behavior upon contact with body fluids. The chemical composition of the bulk material is often significantly different from the surface interfacing with organic tissues. Some materials, such as titanium, undergo surface oxidation, and the mode of preparation or sterilization may result in chemical contamination of the surface [11].

## 3. Structural and Chemical Properties of Surfaces

Numerous* in vitro* experiments and animal studies have shown the importance of the implant surface’s characteristics in the host response [12]. It is known that abutment implant surfaces must present smooth surfaces to favor cell adhesion whereas implant surfaces must be rough to promote osteoblast proliferation [13]. However, the optimal surface topography for implant abutments has yet to be determined [14].

The manufactured surface can be considered one of the factors that will determine the formation of new tissue around the implant. The surface properties of any material will be different from the bulk of the material. The creation of a surface inevitably involves breaking of the chemical bonds that keep the material together. A freshly created surface represents an energetically unstable situation, often referred to as having a high surface energy. When the new surface is exposed to novel environment, the surface energy will rapidly be lowered by binding to and reacting with surrounding molecules. For metals such as titanium, these reactions involve oxygen in the air to form a thin surface layer of oxide. At the same time, the surface characteristics are also strongly influenced by the method of surface preparation, handling and storage. During the preparation of abutment implants, the material surface is subjected to various chemical processes that leave residues on the surface. If the preparation involves elevated temperatures, the surface oxide will grow as a result. Sterilization and storage in sterile packaging are also likely to influence the surface, for example, via the transfer of molecules from the packaging material to the implant surface. The close connection between surface preparation and the resulting surface characteristics means that all aspects of the manufacturing process and ensuing logistics need to be carefully controlled to produce consistent abutment implant surfaces.

**Figure 1:**
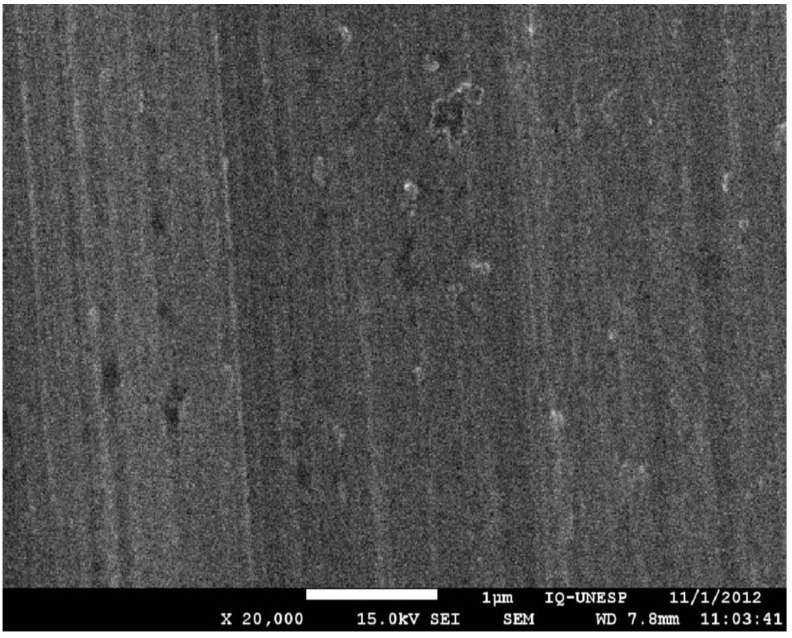
Scanning electron microscopy (magnification 20,000×) of the titanium microstructure.

**Figure 2:**
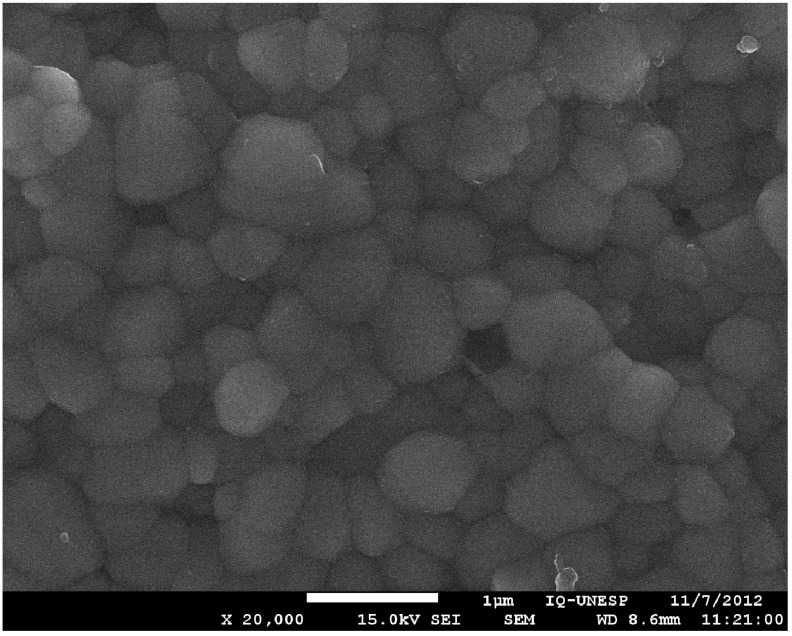
Scanning electron microscopy (magnification 20,000×) of the zirconia microstructure.

A particularly important structural property of dental abutment implants is the surface topography or surface roughness. Figure 1 and Figure 2 show the different topographies of two kinds of surfaces, titanium and zirconia, by means scanning electronic microscopy (SEM).

Depending on the scale being considered, the roughness will be determined by the surface oxide layer or by the bulk material. The surface structure may be completely dominated by the surface oxide layer if it is thick. In other cases, it is determined by a combination of a micrometer-scale rough metal surface covered by a thin oxide layer with nanometer-scale roughness. Whereas it is well established that surface roughness on the micrometer scale plays an important role in cellular reactions, tissue healing and implant fixation [4], the role of surface topography on the nanometer scale has not yet been explored in a systematic manner. The variety of surface characteristics that are possible for abutment implants opens up opportunities for modifying implant surfaces to enhance their biological performance. The clinical abutment implants currently in use display a wide variety of micro-structural and chemical properties. Different mechanical, chemical and optical methods are used to produce abutment implant surfaces with various surface topographies and oxide layers of different thicknesses, crystallinities and compositions.

There are two main kinds of dental abutment implants on the market: titanium and zirconia (or zirconium dioxide, ZrO_2_). Pure titanium or titanium alloys, and to a lesser extent, zirconium, are metals that are often used in direct contact with host tissues. These metallic biomaterials are highly reactive, and on exposure to fluid media or air, quickly develop a layer of titanium dioxide or zirconium dioxide. This layer of dioxide forms a barrier at the interface between the biological medium and the metal structure, determining the degree of biocompatibility and the biological response to the implant. For titanium, the chemical composition of the material is usually the dioxide, TiO_2_, which is a chemically inert, semiconducting material that also exhibits photocatalytic activity in the presence of light of energies equal to or higher than its band-gap energy. These characteristics offer an extensive range of applications in dental implants as prosthetic components. For these reasons, titanium implants have gained widespread attention over recent decades. The surface oxide may also include varying amounts of other substances as impurities. Organic molecules originating from adsorbed molecules from the air, process residues or packaging materials also cover these surfaces. The residues formed on the surfaces may influence their wetting properties and, hence, important interactions such as protein adsorption. The thickness of the surface oxide layer on titanium can vary from a few nanometers to several micrometers depending on the method of preparation and the temperatures involved.

Abutment implants made of ceramic materials can eliminate the problems associated with metal being visible in the peri-implant area, offering important esthetic advantages. The yttria-doped tetragonal zirconia polycrystal (Y-TZP) has become an alternative to alumina as a structural bioceramic because of its significantly higher fracture toughness and strength [15,16]. Y-TZP was first used in orthopedics, allowing new implant designs that were not possible with the more brittle alumina. Biomedical grade Y-TZP exhibits the best mechanical properties of single-phase oxide ceramics, which are the results of phase-transformation toughening to increase its crack-propagation resistance. Zirconia exists in three phases (monoclinic, tetragonal and cubic) according to the temperature [17]. In zirconia, the high strain energy at a crack tip creates T-M (tetragonal-monoclinic) transitions. This crystalline modification is followed by a 4% volumetric expansion that closes the crack [18,19,20]. Y-TZP ceramics can exhibit toughnesses higher than 6 MPa∙pm and strengths higher than 1000 MPa. On the other hand, due to its metastability, Y-TZP is prone to low-temperature degradation (LTD), also referred as aging, in the presence of water. Aging is a progressive transformation from a tetragonal phase to monoclinic, which results in surface modification and microcracking. This process occurs due to the high modulus of elasticity of zirconia, which inevitably influences the performance and reliability of zirconia devices and reduces their lifetime [18]. In orthopedics, clinical reports show that Y-TZP can exhibit progressive degradation even under well-controlled process conditions, which limits its long-term stability. Interestingly, Y-TZP is no longer used in orthopedics, and major companies in this field have switched to alumina-zirconia composites. In dentistry, zirconia is used in the monolithic phase as 3Y-TZP. Polycrystalline tetragonal zirconia stabilized by yttria (3 mol%) results in a ceramic material with high toughness and hardness [21]. In recent years, zirconia dental abutment implants have been favored over titanium implants, especially in the anterior part of the oral cavity, for implant-supported prostheses [22,23] due to their excellent strength and toughness but also due to their esthetic properties, translucency, ability to be colored, the availability of new powders with superior aging resistance and ability to be manufactured by computer-aided design and manufacturing procedures. Even though a few general papers devoted to dental zirconia have underlined the fact that some forms of zirconia are susceptible to aging and that processing conditions can play a critical role in the LTD of zirconia [21], this problem has not received sufficient attention to date.

## 4. Problems of Dental Abutment Implant Surfaces

Corrosion is the deterioration a metal undergoes as a result of interactions with the surrounding medium (electrochemical attack), which causes the release of ions into the environment. It is important to mention that no metal or alloy is entirely inert* in vivo*. And corrosion phenomena at the surface interface are particularly important in the evolution of both dental and orthopedic implants and are possible causes of implant failure after an initial success. The degradation of a metallic implant is undesirable because it negatively alters the structural integrity of the implant [24]. Treatments of Ti-based implants give rise to an outer rutile layer that improves corrosion resistance and reduces the friction coefficient of rubbing contact [25,26]. By definition, rutile is a mineral composed primarily of titanium dioxide, TiO_2._ These surfaces improve osteoblast adhesion *in vitro* and increase the percentage of bone-to-implant contact* in vivo* [27,28]. Rutile debris are expected to arise from these modified surfaces after long-term functional loading. Valles *et al.* [29] investigated whether human osteoblasts were able to absorb rutile particles compared with their intake of titanium particles. The dry rutile and titanium particles used in the experiment were different in size (rutile of 0.9–1.6 mm in diameter and commercially pure titanium (Ti) particles of 0.20 mm in diameter). Cell treatments were performed with equivalent amounts of each type of material. In principle, the osteoblasts should have received a substantially higher number of rutile particles than titanium particles. However, examination of the particles as suspensions in the culture media before being applied to the cells revealed the formation of micrometric aggregates in both cases. Therefore, the cells were actually in contact with agglomerates of rutile or titanium particles of a similar size range rather than with individual particles, and the rutile particles induced a lower response* in vitro*, as defined by their ability to induce the secretion of inflammatory cytokines (TNF-a, IL-6 and IL-1b) in macrophage cultures of different sources. Other authors have noted that sub-micrometric dry alumina particles aggregate to the same extent as micrometric dry titanium particles and have previously detected agglomerations of other kinds of particles in culture media. Treatment with titanium or rutile particles does not result in osteoblast death. Similar doses of titanium particles are not cytotoxic for human osteoblast-like MG-63 cells [30], but they severely decreased the viability of rat osteoblasts [31], suggesting that species-specific characteristics modulate the sensitivity of osteoblasts to particles generated by wear. These works collectively show that corrosion is not a local problem because the particles produced as a result can migrate to distant sites. If these particles can reduce the viability of osteoblasts in animals, it is possible that they could also cause chronic inflammation because the macrophages that phagocytose these particles cannot digest them, so they get released in the middle of their transport. Other macrophages will phagocytose these particles again, and the cycle will repeat. Interestingly, treating human primary macrophages with Ti particles releases much higher levels of inflammatory cytokines (TNF-a, IL-6 and IL-1b) than rutile, which only stimulates marginally detectable levels of secretion. These results support the higher biocompatibility of titanium-based implants modified to create an outer layer of rutile on their surfaces.

In relation to zirconia, most of the research on zirconia dental ceramics today focuses on the mechanical properties of the devices [32], their fatigue resistance [33] and surface modifications [34] that could enhance bone in-growth and, in cases of dental implants, reduce bacterial adhesion and favor the growth of epithelial cells on abutment surfaces [35]. Recently, Chevalier *et al.* [18] evaluated the resistance of biomedical-grade yttria-stabilized zirconia samples coated with a porous zirconia layer that was processed via two slightly different routes to environmental degradation. In one group, the porous surface was coated onto a pre-sintered ceramic piece. In the other group, the porous surface was coated and sintered together with the ceramic piece. The results showed that the two groups exhibited totally different degrees of LTD resistance. With the other coating process, we would expect full transformation of the porous layer after 5 years* in vivo* in the worst-case scenario. Standard steam sterilization at 134 °C for just 1 h would lead to a significant transformation of this layer. The only change was the sequence by which the porous surface was sprayed onto the surface. This result was very important because it confirmed the strong variability of 3Y-TZP with regard to LTD resistance and the critical role of the manufacturing process. It is therefore essential to more systematically evaluate the resistance of any new dental device dedicated to clinical use to LTD to avoid critical issues such as those encountered in orthopedics some years ago. The search for aging-resistant zirconia and standardized LTD evaluations should be a priority in implant research to ensure the long-term success of zirconia as a dental material.

## 5. Dental Abutment Implants and Periodontal Tissue

Despite the widespread use of titanium and the substantially growing research on the development of new surfaces and/or modifications of existing surfaces, a detailed understanding of the relationship among surfaces, cells and bacteria adhesion is still lacking. The soft tissue around dental implants serves as a protective barrier between the oral environment and the underlying peri-implant bone, and one factor proposed to be of importance for the long-term success of implant therapy is the development of a good seal between the abutment and soft-tissue [36]. Modifications of abutment implants to improve esthetics should not be made at the expense of biological compatibility. Placement of an abutment is followed by a sequence of biological events: covering the surfaces with a pellicle of proteins and glycoproteins derived from saliva and gingival crevicular fluid; the adherence, migration and proliferation of cells; and the secretion of microbial products [37]. The composition, as well as the configuration and density, of the proteins in the pellicle, are largely dependent on the physical and chemical nature of the underlying surface. It follows that the properties of the surface influence bacterial adhesion through pellicle protein adsorption and the adherence, migration and proliferation of cells. Improved understanding of these sequences would aid in the selection of an optimal surface texture.

In relation to cell attachment, smooth, turned titanium, nanoporous TiO_2_-coated and anodized Ca^2+^-modified surfaces have all been shown to be suitable for soft-tissue healing [38,39]. Fröjd* et al.* [39] investigated how different implant surfaces (turned titanium, sol-gel nanoporous TiO_2_-coated surfaces and anodized Ca^2+^-modified surfaces) affect biofilm formation by two early colonizers of the oral cavity. Nano-topographical modification of smooth titanium surfaces did not cause significantly greater bacterial adhesion and biofilm formation* in vitro* than turned surfaces or surfaces treated with Ca^2+^ incorporation during anodic oxidation. In the presence of saliva, adhesion increased by more than ten-fold compared with without saliva, and yet, no differences were observed among the surfaces. These data suggest that modification with sol-gel-derived nanoporous TiO_2_, which has been shown to improve soft-tissue healing* in vivo*, does not lead to greater bacterial adhesion and initial biofilm formation by the two commensal species tested compared with other surfaces [40]. However, it cannot be discounted that greater differences in biofilm formation on the different surfaces could be observed over a longer time period in the presence of other bacterial species. According to Abrahamsson* et al.* [36], abutments made of titanium or highly sintered aluminum-based ceramic (Al_2_O_3_) allowed the formation of a mucosal attachment that included epithelial and connective tissue that were approximately 2 and 1.5 mm thick, respectively. In contrast, with porcelain dental implants, no mucosal attachment formed at the abutment level; instead, the soft tissue margin receded, and bone resorption occurred. The mucosal barrier was thus partially established at the fixture portion of the implant. Mustafa* et al* [14]*.* investigated the attachment and proliferation of human oral fibroblasts in densely sintered aluminum oxide specimens. The authors concluded that the initial attachment and spreading of human gingival fibroblasts were influenced by the surface texture of the ceramic abutments. Fibroblasts spread and grew effectively on sintered surfaces that had their roughness (Sa) increased to 0.34 mm by milling. Other studies have shown statistically significant differences between peri-implant soft tissues around titanium and zirconium oxide healing caps, with an overall lower inflammatory level in tissues surrounding the latter [41]. To understand these results, it is necessary to understand the relationship between periodontal tissue and prosthetic components of implants. The biological extension around natural teeth has been reported to be approximately 2 mm, 1 mm of which corresponds to epithelial attachment mediated by the junctional epithelial (JE) and 1 mm of which corresponds to gingival connective tissue attachment [42]. Several studies have described that the peri-implant JE is approximately 2 mm long [43]. This value can usually be increased because conventional implant surfaces cannot deter the formation of a “long” epithelial attachment. However, as long as the JE stays restricted to the region of the prosthetic components and not the implants, it will not cause damage. In other studies, the peri-implant epithelium (PIE) appeared to lean on the abutment implant, but was structurally very different from the JE, showing slower cell proliferation and no evidence of direct adhesion on the implant surface [44]. Poor adhesion of the PIE may contribute to the formation of inflammatory lesions and bone loss around the implants, which has become a common clinical problem [45,46]. It is possible that low PIE adhesion allows for apical migration of plaque biofilms and could, therefore, directly explain the inflammation and bone loss around bone-level dental implants.

## 6. Dental Abutments Implants and Bacteria Adhesion

It is known that bacterial plaque plays a prominent role as an etiologic factor in implant loss after osseointegration due to the presence of high levels of bacteria in peri-implant sites [47,48,49]. As observed for teeth, the microorganisms need to interact with the abutment implant surface for the formation and growth of a biofilm. Firstly, this interaction occurs through non-specific physicochemical mechanisms. Bacterial adhesion involves the superficial free energies and interaction surfaces theory in which adhesion is regarded as the interaction of the van der Waals forces and electrostatic phenomena. After the interactions of the biomaterial surfaces with biological systems* in vitro* or* in vivo*, the proteins present in the biological medium immediately coat the surfaces [50]. In sequence, the acquired salivary pellicle formation takes place as the first step in biofilm formation. Early colonizers create an environment that favors late colonizers. Several studies have suggested that some restorative materials may have antibacterial activity, while others may induce bacterial growth [51,52,53,54]. With regard to the influence of surface roughness on biofilm formation, previous reports have shown that protein adsorption and bacterial adhesion* in vivo* appear to require a threshold surface roughness of 0.2 μm [55,56]. Burgers* et al.* [57] evaluated the initial biofilm formation on different titanium surfaces* in vitro* and* in vivo* and correlated these findings with different surface properties. Before biofilm formation, the authors determined the surface roughness and the surface free energy of the samples. Their results showed that the initial bacterial adhesion to differently textured titanium surfaces was primarily influenced by surface roughness values. According to these authors, the parts of an implant that are exposed to the oral cavity should be polished to prevent plaque accumulation. Another crucial element that directly influences bacterial adhesion is surface hydrophobicity [58] because a very hydrophobic surface may prevent water from wetting the available surface, and thus prevent protein interaction with it. Alternatively, an increase in the surface hydrophilicity may reduce the hydrophobic interaction between proteins and the surface, causing a lower adsorption affinity.

From the literature, it is still uncertain what the ideal abutment implant surface should be to reduce bacterial adhesion [52,53,54,59,60,61,62,63,64] (Table 1).

Some* in vitro* and* in vivo* studies have confirmed differences in biofilm formation among different types of materials. According to some authors, the biomaterial-related properties of zirconia are more advantageous than titanium. Bacterial adhesion has been shown to be satisfactorily low in zirconia restorations, which is important in maintaining periodontal health [52]. Scarano* et al.* [23] studied discs attached to a device worn intraorally and reported a degree of coverage by bacteria of 12.1% on zirconia discs compared with 19.3% on titanium discs. This difference was attributed to the fact that zirconia had a lower electrical conductivity. Rimondini* et al.* [59] confirmed these results in an* in vivo* study that showed that zirconia surfaces accumulated fewer bacteria than titanium due to their chemical properties after correcting for the standard roughnesses of surfaces for all of the samples of the same group but with different materials. In concordance, other authors evaluated biofilm formation on various types of titanium and zirconia abutment surfaces* in vivo* and concluded that oral biofilm accumulation was lower on zirconia surfaces compared with titanium surfaces [52]. At the same time, inflammatory infiltration, microvessel densities and vascular endothelial growth factor expression were found to be higher around titanium caps than zirconia caps [65]. In addition, patients have reported allergic reactions and sensitivities to titanium [39,66]. The material composition of transgingival implant components appears to influence the formation of epithelial attachment. The shape and profile of the implants are able to guide gingival contouring and, together with the color of the material, strongly influence the final esthetic results of dental implant restorations. Zirconia can be suitable for making implant abutments, but more clinical trials and mechanical testing are necessary for a fuller understanding of the behavior of zirconia abutments over a long time period.

**Table 1:** Studies presenting data on microbiology associated with abutments dental implants.

Authors	Kinds of study	Surface studied	Predominant microbes	Methods used	Results
Rimondini* et al.* [59] 2002	*In vivo*	Titanium and Zirconia	*S. mutans, S. sanguis, A. viscosus, A. naeslundii,* and* P. gingivalis*	Quantification of bacteria	Zirconia accumulates fewer bacteria than titanium.
Al-Ahmad* et al.* [52] 2010	*In vivo*	Machined Ti, modified Ti, modified Zr machined alumina-toughened Zr, sandblasted alumina-toughened Zr, machined Zr, Ti, Zr	*S. spp., V. spp., F. nucleatum,* and *A. naeslundii*	Fluorescence* in situ* hybridization and confocal laser scanning microscopy	There was no difference in bacteria adhesion between titanium and zirconia
van Brakel* et al.* [54] 2011	*In vivo*	Titanium and Zirconia	*A. actinomycetemcomitans, P. gingivalis, P. intermedia, T. forsythia, P. micros, F. nucleatum, T. denticola*	Quantification by means real-time PCR	There was no difference in bacteria adhesion between titanium and zirconia
Lee* et al.* [60] 2011	*In vitro*	Titanium and Zirconia	*S. sanguis*	Quantification by means scanning electron microscope, crystal violet staining and measurement of fluorescence intensity	There was no difference in bacteria adhesion between titanium and zirconia
Salihoglu* et al.* [61] 2011	*In vivo*	Titanium and Zirconia	*A. actinomycetemcomitans, P. gingivalis*	Bacterial detection and quantification by means real-time PCR	There was no difference in bacteria adhesion between titanium and zirconia
Al Radha* et al.* [62] 2012	*In vitro*	Titanium, Zirconia, Titanium blasted with zirconia, Titanium blasted with zirconia/acid etched	*P. nigrescens, S. mitis*	Fluorescence microscopy; the area covered by bacteria was calculated using Image-J software	Zirconia and Titanium blasted with zirconia showed superior effect reducing the adhesion of bacteria
Yamane*et al.* [63] 2013	*In situ*	Titanium, gold-platinum alloy, zirconia, alumina, and hydroxyapatite	*S. mutans*	Quantification by means PCR	There was no difference in bacteria adhered and the tested materials
Oliveira1*et al.* [64] 2012	*In vivo*	Titanium and Zirconia	*A. actinomycetemcomitans, P. gingivalis*	Quantification by means real-time PCR	There was no difference in bacteria adhesion between titanium and zirconia
Do Nascimento* et al.* [53] 2013	*In vitro*	Machined titanium, cast titanium and zirconia abutments	*F. nucleatum, N. mucosa, P. aeruginosa, P. anaerobios, S. aureus, S. gordonii, S. parasanguinis, T. forsythia*	Biofilm percentage was calculated using the relation between biofilm area and total surface area of specimens.	Zirconia accumulates fewer bacteria than titanium

In recent years, some new studies have attempted to compare the adhesion of aerobic bacteria (*in vitro*) and anaerobic bacteria (*in situ*) on titanium and zirconia abutments, and many of them have found no differences in the quantity of cells adhered to different surfaces [53,54,67]. Salihoglu* et al.* [61] compared zirconium dioxide (zirconia) and titanium alloys with respect to the adhesion and colonization of two periodontal pathogens on both hard surfaces and on soft tissues* in vivo*. The results showed no statistically significant differences in probing depths, number of DNA copies of *A. actinomycetemcomitans* or* P. gingivalis*, and total bacteria counts between titanium alloys and zirconium oxide surfaces and between the biopsy specimens obtained from their buccal gingival. With respect to the surface free energy, zirconia abutments showed lower surface free energies than titanium abutments. According to these authors, zirconia surfaces have comparable properties to titanium alloy surfaces with respect to the adhesion and colonization of two periodontal pathogens on both hard surfaces and in soft tissues [61]. Therefore, future research should focus on improving epithelial attachment on implants and reducing biofilm adhesion, especially on different abutments.

## 7. Conclusions

Implant surfaces are composed of chemical elements, which may degrade under different temperatures or suffer damage from the forces applied to them. The release of such chemical elements may result in inflammation of the periodontal tissue. At the same time, the surface characteristics may be altered, thus favoring biofilm development, which will further increase inflammation. If not treated, this inflammation may cause the loss of the implant. Today, it is already known that implant surfaces should be modified not just to reduce microbial adhesion but also to reduce the chemical elements released by the surfaces over time. Further research is necessary to create an abutment surface that can achieve all of these goals, which is currently the biggest challenge in oral rehabilitation with dental implants.
